# Analysis of Spatiotemporal Changes in Jasmonic Acid, Salicylic Acid, and Indole-3-Acetic Acid in Tea Plants Based on Silylation and Gas Chromatography-Mass Spectrometry

**DOI:** 10.3390/biology15201793

**Published:** 2026-10-09

**Authors:** Chenqi Sun, Kunzhu Hu, Lei Bian, Zongxiu Luo, Zhaoqun Li, Chunli Xiu, Nanxia Fu, Zongmao Chen, Yu Gao, Xiaoming Cai

**Affiliations:** 1 Key Laboratory of Soybean Diseases and Pests Control in Ministry of Agriculture and Rural Affairs, College of Plant Protection, Jilin Agricultural University, Changchun 130118, China; sun446140168@163.com; 2 Key Laboratory of Biology, Genetics and Breeding of Special Economic Animals and Plants, Ministry of Agriculture and Rural Affairs, Tea Research Institute, Chinese Academy of Agricultural Science, Hangzhou 310008, China

**Keywords:** jasmonic acid, salicylic acid, indole-3-acetic acid, silylation derivatization, gas chromatography–mass spectrometry, metabolism quenching, spatiotemporal change

## Abstract

Phytohormones play a vital role in plant responses to biotic stresses. Although phytohormone determination mainly employs ultra-high-performance liquid chromatography–tandem mass spectrometry (UHPLC-MS/MS), its high costs and technical requirements limit utility, whereas gas chromatography–mass spectrometry (GC-MS) is more widely used. To determine jasmonic acid (JA), salicylic acid (SA), and indole-3-acetic acid (IAA) in tea plants, a GC-MS method was developed, including selection of silylating reagents and establishment of metabolism quenching and purification methods. SA, JA, and IAA contents in tea leaves increased significantly after storage at −80 °C or extraction at room temperature. These increases were prevented by immersing samples in a 95 °C water bath. Quantification limits for SA, JA, and IAA were 0.020 ng mg^−1^, 0.015 ng g^−1^, and 0.015 ng g^−1^, similar to or exceeding those obtained by UHPLC-MS/MS. Recoveries and RSD were 93.1–98.9% and 2.1–9.9%, respectively. Only 10 mg of tea leaves was sufficient for simultaneous analysis of the three phytohormones. The JA response to geometrids was strong compared with the SA response, whereas the opposite trend was observed for leafhoppers. The systemic responses of JA and SA were rapid, similar to the local response. These results indicate that the proposed method is sensitive and reliable.

## Simple Summary

Tea is one of the most popular drinks in the world, but tea plants are often attacked by insect pests, reducing yield and quality. When damaged, they produce chemical signals that help them defend themselves. Three of the most important of these signals are jasmonic acid (JA), salicylic acid (SA), and indole-3-acetic acid. Measuring these signals accurately is difficult and expensive with current methods. In this study, we developed a simpler and cheaper method using gas chromatography–mass spectrometry to measure all three signals at the same time in tea plants. We also found that these signals change when tea leaves are frozen or kept at room temperature, and that briefly placing the leaves in hot water prevents these unwanted changes, making the measurements more reliable. Using our method, we showed that tea plants respond differently to two major pests: caterpillars that chew leaves mainly lead to increased JA accumulation, while leafhoppers that suck plant sap mainly lead to increased SA accumulation. Our method requires only 10 mg of tea leaves and could help scientists better understand how tea plants protect themselves, supporting the development of safer and more sustainable pest control in tea farming.

## 1. Introduction

Phytohormones are structurally diverse small molecular compounds that act as chemical messengers between cells. They play an important role in plant growth, development, and resistance to biotic and abiotic stresses [1]. Specifically, jasmonic acid (JA) and salicylic acid (SA) are central components of signaling pathways that activate and precisely modulate defense mechanisms in response to herbivorous insects and pathogens. Typically, the JA signaling pathway regulates plant responses and resistance to leaf-chewing herbivores, whereas the SA signaling pathway plays a crucial role in the response to phloem-feeding herbivores [2,3,4]. Moreover, multiple phytohormones act through signaling networks and often influence the same biological processes via additive, synergistic, or antagonistic effects. The synergistic and antagonistic interactions between JA and SA significantly enhance or weaken plant defenses [3,4,5,6]. Additionally, JA or SA could interact with growth phytohormones to maintain a balance between plant growth and defence [7,8,9]. For example, the cross-talk between SA and indole-3-acetic acid (IAA), which plays an important role in plant growth and development processes such as cell elongation and fruit development [10,11], mediates plant defense against sucking pests and pathogen infestation [12,13]. Therefore, to more comprehensively characterize molecular mechanisms and phytohormone interactions in complex signaling networks, their diversification and spatiotemporal differences should be investigated. This type of analysis requires the ability to accurately and efficiently examine multiple phytohormones simultaneously. Moreover, downscaling sample requirements can enhance the spatial resolution of phytohormone analyses. However, low phytohormone contents in plant samples and their complex matrices and structural diversity make it difficult to develop improved methods for the simultaneous examination of multiple phytohormones.

To date, a number of analytical techniques have been developed for analyzing phytohormones, each with its own advantages and disadvantages. For example, chromatographic methods, including gas chromatography (GC) and liquid chromatography (LC) combined with different types of mass spectrometry (MS), are highly sensitive and excellent for separating compounds, making them appropriate for simultaneously analyzing multiple hormones in complex matrices [14,15,16,17]. Ultra-high-performance liquid chromatography–tandem mass spectrometry (UHPLC-MS/MS) methods are often used to detect phytohormones because many plant hormones are non-volatile compounds [18]. Tandem MS involving triple quadrupole instruments operated in the multiple reaction monitoring mode with fast duty cycles and decreased chemical noise is highly sensitive [19]. However, the high costs and technical requirements of UHPLC-MS/MS systems limit their utility. Compared with UHPLC-MS/MS, GC-MS is more widely used. Generally, GC-MS methods may benefit from the conversion of phytohormones to their more volatile, thermally stable derivatives, with the most comprehensive derivatization-based methods requiring silylating agents. In addition, considering their sensitivity, GC-MS and LC-MS methods depend on an efficient purification procedure because MS analyses of plant samples are substantially influenced by other compounds present in tissues that can suppress or enhance analyte ionization [20,21].

Globally, tea is the most popular beverage after water, with considerable health benefits for humans. Hence, tea is one of the most important and traditional economically important crops in many developing countries in Asia, Africa, and Latin America between latitudes 41° N and 16° S [22,23]. Previous studies showed that JA, SA, IAA, and other signal pathways regulate tea plant defense responses to various pests [24,25,26,27,28]. Notably, JA and SA are the core signaling compounds; the synergistic or antagonistic interaction between JA and SA pathways modulates the tea plant metabolome and influences tea plant resistance to herbivores [6]. However, the mechanism regulating the phytohormone signaling network remains to be more thoroughly investigated. Moreover, tea leaves are a rich source of secondary metabolites, which may interfere with the isolation and identification of phytohormones.

In the present study, we used a GC-MS system to develop a highly sensitive and reliable method for analyzing JA, SA, and IAA in tea plants. A simple purification procedure and a reliable silylation-based derivatization were completed before the GC-MS analysis. Additionally, a metabolism-quenching method was developed to ensure stable storage and simplified analyses. Finally, the developed method was used to determine the spatiotemporal changes in JA, SA, and IAA contents in tea plants damaged by tea green leafhoppers (*Empoasca onukii*) with piercing–sucking mouthparts and gray tea geometrids (*Ectropis grisescens*) with chewing mouthparts, which are the two most important pests of tea plants in China [23]. Our method may be useful for studies on tea plant stress-related responses involving these phytohormones.

## 2. Materials and Methods

### 2.1. Reagents

SA (98%), JA (98%), and IAA (98%) were purchased from Shanghai Macklin Biochemical Technology Co., Ltd. (Shanghai, China), whereas *N*,*O*-bis(trimethylsilyl)trifluoroacetamide (BSTFA, 98%), *N*-(t-butyldimethylsilyl)-*N*-methyltrifluoroacetamide (MTBSTFA, 99% with 1% *t*-BDMCS), and MTBSTFA (97%) were purchased from Sigma-Aldrich (Shanghai) Trading Co., Ltd. (Shanghai, China). Additionally, *N*-propylethylenediamine (PSA), graphitized carbon black (GCB), and multi-walled carbon nanotube (MWCNT, 10–20 nm outer diameter) were purchased from Beijing Mreda Technology Co., Ltd. (Beijing, China). Standard JA, SA, and IAA solutions were prepared in ethyl acetate. A mixture comprising SA (8 ng µL^−1^), JA (8 ng mL^−1^), and IAA (8 ng mL^−1^) and SA solutions at different concentrations were used for optimizing the derivatization and sample purification.

### 2.2. Plant and Insect Materials

One-year-old tea plants (*Camellia sinensis* ‘Longjing 43’) were transplanted individually into plastic pots (25 cm height, 20 cm diameter) filled with potting soil and then maintained in a climate chamber set at 25 ± 2 °C with 60–75% relative humidity (RH) and a 14 h light (05:00–19:00):10 h dark cycle. One year later, the healthy and insect-free tea plants, which were approximately 25 cm tall, were used for experiments. Gray tea geometrid (*E. grisescens*) larvae and tea green leafhopper (*E. onukii*) adults were obtained from a plantation (Shaoxing Royal Tea Village Co., Ltd.) in Shaoxing, China. Geometrids and leafhoppers were maintained in a growth chamber set at 25 ± 2 °C with 70–80% RH and a 14 h light (05:00–19:00):10 h dark cycle, and reared on fresh tea leaves (cv. Longjing 43) for at least two generations.

### 2.3. Method Development: Derivatization

The sensitivity, stability, and contamination of three common silylating agents were compared. Specifically, 50 µL SA standard solution (8 ng µL^−1^) was reacted with 50 µL BSTFA, MTBSTFA (97%), or MTBSTFA (99% with 1% *t*-BDMCS) at 90 °C for 30 min (*n* = 4). Samples were stored at room temperature (25 ± 2 °C) and analyzed at 0, 24, and 96 h post-reaction. Next, the derivatization of MTBSTFA (97%) was optimized. Reaction conditions were as follows: 50 °C for 30 min, 75 °C for 30 min, 75 °C for 50 min, 90 °C for 30 min, 90 °C for 50 min, and 120 °C for 30 min. In this test, a 50 µL mixture comprising SA, JA, and IAA was reacted with 50 µL MTBSTFA (*n* = 4). The reaction efficiency of this system, in which the MTBSTFA-to-sample volume ratio was 1:8, was analyzed using SA solutions at different concentrations (500.0, 125.0, 25.0, 2.5, 0.6, and 0.1 ng µL^−1^) at 90 °C for 30 min (*n* = 4). The reaction efficiency was also analyzed using 2.5 ng µL^−1^ SA solution at three MTBSTFA-to-sample volume ratios (1:1, 1:4, and 1:8) at 90 °C for 30 min (*n* = 4).

### 2.4. Method Development: Sample Extraction

Freshly collected leaves were immediately ground to a powder in liquid nitrogen, after which 200 mg of powdered sample was added to a grinding tube containing three steel beads for homogenization (60 Hz for 180 s) using a multi-sample tissue grinder (Tissuelyser-24, Shanghai, China). Before homogenization, the grinding tube, steel beads, and related adapters were treated with liquid nitrogen. To decrease polar impurities, ethyl acetate was selected as the extraction solvent. After homogenization, 1 mL ethyl acetate was immediately added to the sample, after which the mixture was subjected to an ultrasound extraction in an ice bath for 20 min. The extract (800 µL) was centrifuged at 10,000 r min^−1^ for 5 min at 4 °C and then 600 µL supernatant was collected for the subsequent purification.

### 2.5. Method Development: Sample Purification

A two-step cleanup procedure involving miniaturized liquid–liquid extraction (MLLE) and quick, easy, cheap, effective, rugged, and safe (QuEChERS) methods was established. MLLE was completed using a 1.5 mL injection vial. The SA, JA, and IAA mixture (600 µL) was used to determine recovery rates at two temperatures (0 and 25 °C, *n* = 4) and two water volumes (100 and 400 µL, *n* = 4). For the temperature test, 400 µL ultrapure water was used. For the water volume test, the temperature was set at 0 °C. For the 0 °C test, the injection vial was placed in a 5 mL centrifuge tube that was filled with ice. Injection vials or centrifuge tubes were vortexed at 2200 r min^−1^ for 10 min and then left undisturbed for 5 min. Next, 200 µL of the upper layer of the standard solution was collected for derivatization, whereas 400 µL of the upper layer of the extract was collected for QuEChERS. For the QuEChERS method, three adsorption materials (GCB, PSA, and MWCNT) were evaluated in terms of their phytohormone adsorption capacity (*n* = 4). The SA, JA, and IAA mixture (400 µL) was used along with 12 mg of adsorption material. The mixture was vortexed at 2200 r min^−1^ for 3 min and then centrifuged at 5000 r min^−1^ for 5 min, after which 200 µL supernatant was reacted with 50 µL MTBSTFA.

Considering that the silylating agents are sensitive to water, two methods for dehydrating the extract were assessed. The tested sample was 8 ng µL^−1^ SA standard solution containing 2% ultrapure water. First, a nitrogen-blowing treatment (1.2 L min^−1^, 25 °C) was tested using 200 µL samples. The duration of the nitrogen-blowing treatment was set at 30, 45, and 90 s (*n* = 4), after which 200 µL ethyl acetate was added immediately, and the solution was reacted with 50 µL MTBSTFA. Second, anhydrous magnesium sulfate (0.1 g) and anhydrous sodium sulfate (0.1 and 0.4 g) were added to 400 µL samples (*n* = 4) as dehydrating agents. The subsequent process was the same as that used for QuEChERS. Finally, 200 µL supernatant was reacted with 50 µL MTBSTFA. For both tests, 200 µL of water-free SA solution (8 ng µL^−1^) was reacted directly with 50 µL MTBSTFA to calculate the recovery rate.

### 2.6. Method Development: Sample Pre-Treatment for Metabolism Quenching

To prevent changes in phytohormone contents during storage and extraction, a metabolism-quenching method was established in which freshly collected samples were immediately immersed in hot water. The effects of this method were tested once during the storage stage and twice in the extraction stage. To ensure sample consistency across the four pre-treatments (80 °C for 10 min, 95 °C for 10 min, 100 °C for 15 min, and non-enzymatic quenching as a control), sufficient fresh tea leaves were chopped, mixed thoroughly, and then divided into four equal portions for the respective pre-treatments. The pre-treated samples were immediately frozen in liquid nitrogen and then stored at −80 °C or used for extract preparation. In the storage stage, the four pre-treated samples were removed from the freezer (−80 °C) after 0, 3, 7, 11, and 15 days and then extracted at 0 °C as described above. At each time point, four 200 mg subsamples were taken from each pre-treated sample, and each subsample was independently extracted as a replicate. In the first extraction-stage test, each of the three pre-treated samples (80 °C for 10 min, 95 °C for 10 min, and non-enzymatic quenching as a control) was subjected to two extraction conditions: extraction at 0 °C as described above, and extraction at room temperature after being left for 20 min and then homogenized using a grinder. For each extraction condition, one 200 mg subsample was taken from each pre-treated sample and independently extracted, yielding four replicates per condition. In the second test during the extraction stage, control samples were extracted at room temperature and 0 °C, while metabolism-quenched samples (95 °C for 10 min) were extracted at room temperature. For the above three treatments, one 200 mg subsample was taken from each pre-treated sample and independently extracted, yielding four replicates per condition. For the extraction at room temperature, homogenization, ultrasound extraction, and centrifugation were all completed at room temperature. All samples were purified and derivatized according to the optimized method.

### 2.7. Method Development: Instrumental Analysis

Samples were analyzed using a GC-MS system (QP2010, Shimadzu, Kyoto, Japan) equipped with a DB-5 MS capillary column (60 m × 0.25 mm, 0.25 µm film thickness; Agilent J&W, Folsom, USA). Helium served as the carrier gas, with a flow rate of 1.0 mL min^−1^. The initial oven temperature was set at 45 °C and held for 5 min before being increased to 310 °C at 7 °C min^−1^ and then held for 6 min. Ionization was completed at 70 eV and 250 °C. Samples (1 µL) were injected in the automatic splitless mode. The selective ion monitoring mode was used for high sensitivity and selectivity. The monitored characteristic ions of JA, SA, and IAA are listed in Table S1.

### 2.8. Method Development: Method Validation

Detection and quantification limits were defined as the concentrations corresponding to a signal 3 and 10 times higher than the standard deviation of the baseline noise (*n* = 7), respectively. Calibration curve concentrations (*n* = 3) ranged over four orders of magnitude, with the lowest concentrations close to the respective quantification limits. All calibration samples were spiked with 4 ng of ethyl decanoate as an internal standard after derivatization. Relative calibration curves were established by calculating the analyte-to-internal standard abundance and mass ratios. Accuracy and precision were determined using the addition method. The collected non-spiked control was immediately placed in hot water (95 °C) for 10 min and then stored at −80 °C. SA, JA, and IAA contents in the non-spiked control were 8.35 ± 0.09 ng mg^−1^, 2.54 ± 0.04 ng g^−1^, and 1.48 ± 0.02 ng g^−1^, respectively. The three spiked levels of SA, JA, and IAA were 1/3, 2, and 6 times the basal levels, respectively (*n* = 7). Inter-day values were obtained over 3 consecutive days.

### 2.9. Analysis of Spatiotemporal Changes in JA, SA, and IAA Contents in Tea Plants

To measure local and systemic phytohormone changes in tea plants in response to herbivorous insects, 10 s-instar geometrid larvae or 20 third-instar leafhopper nymphs were confined to the second leaf below the bud using two plastic Petri dishes (60 mm diameter) with aeration holes. Petri dishes were secured using parafilm. The first and third leaves below the damaged leaf, which were used to evaluate the systemic response, were placed in empty Petri dishes. Local and systemic phytohormone changes were evaluated at 0, 2, 6, and 24 h post-infestation. Uninfested plants with the corresponding leaves placed in empty Petri dishes at the same time as the infested plants were used as controls. Geometrid- and leafhopper-infested tea plants and control tea plants were maintained in three well-ventilated rooms under the same conditions (25 ± 2 °C, 60–75% RH, and a 14 h light (05:00–19:00):10 h dark cycle), with each room containing only one treatment. At each sampling time point, four tea plants per treatment were used as biological replicates, and three leaves (damaged, adjacent undamaged, and distal undamaged) were simultaneously collected from each plant. Collected samples were immediately immersed in a 95 °C water bath for 10 min and then either stored at −80 °C or used immediately for extract preparation. Samples were purified and derivatized using the optimized method. SA, JA, and IAA contents were quantified using the relative calibration curves.

### 2.10. Statistical Analyses

Statistical analyses were performed using SAS v8.2 (SAS Institute, Cary, NC, USA). For method development in derivatization, the effects of silylating reagents, storage time, reaction temperature/time, SA concentration, and reagent-to-sample ratio on the derivative response were analyzed separately by one-way analysis of variance (ANOVA) followed by Tukey’s multiple range test. For method development in sample purification, the effects of MLLE temperature and water volume on the recovery rate were analyzed separately using unpaired *t*-tests, whereas the effects of QuEChERS adsorption materials, nitrogen blowing time, and dehydrating agents on the recovery rate were each analyzed by one-way ANOVA followed by Tukey’s multiple range test. In the storage-stage test of metabolism quenching, the effects of the pre-treatments on JA, SA, and IAA contents during storage at −80 °C were analyzed separately by one-way ANOVA followed by Tukey’s multiple range test. In the two extraction-stage tests of metabolism quenching, the JA, SA, and IAA contents of the pre-treated samples extracted at the two temperatures were compared separately using unpaired *t*-tests or one-way ANOVA followed by Tukey’s multiple range test, depending on the number of treatments. To investigate the spatiotemporal changes in JA, SA, and IAA in damaged tea plants, the contents of the three phytohormones in leaves at the same position were compared separately between damaged and undamaged plants using unpaired *t*-tests, whereas the contents among different leaves of the same treated plants were compared separately using one-way ANOVA followed by Tukey’s multiple range test.

## 3. Results and Discussion

### 3.1. Optimization of Derivatization

**Figure 1:**
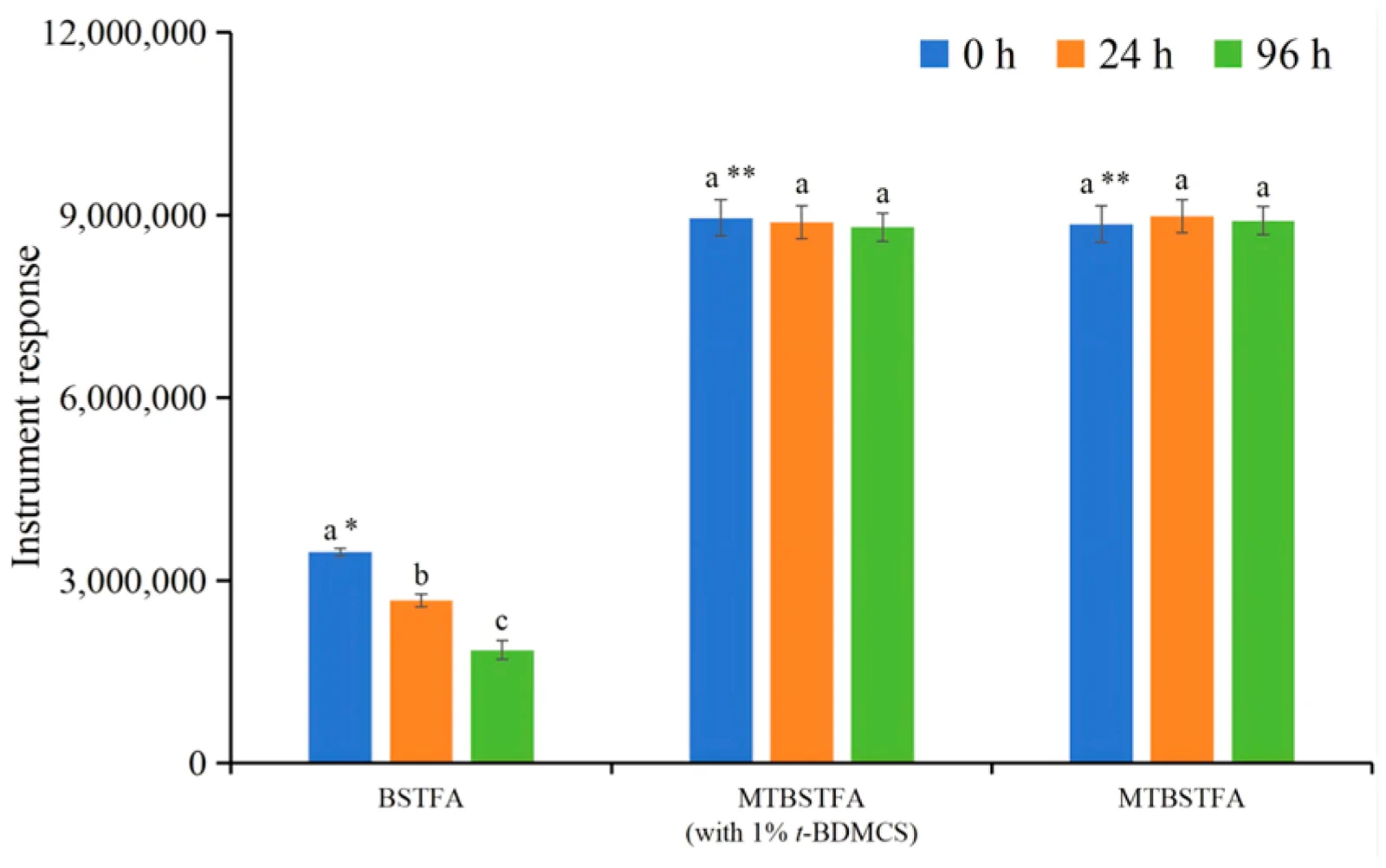
Sensitivity and stability of SA derivatives from BSTFA and MTBSTFA. SA standard solution (8 ng µL^−1^, 50 µL) was reacted with 50 µL BSTFA and MTBSTFA at 90 °C for 30 min. Different letters indicate significant differences among storage times, and different numbers of asterisks indicate significant differences among silylating reagents (Tukey’s test, *p* < 0.05). Data are presented as the mean ± standard error (*n* = 4).

Three silylating agents, including BSTFA (98%), MTBSTFA (99% with 1% *t*-BDMCS) and MTBSTFA (97%), were tested using SA. MTBSTFA produces TBDMS (tert-butyldimethylsilyl) derivatives, whereas BSTFA yields TMS (trimethylsilyl) derivatives. The MS response for TBDMS derivatives was 2.6-fold higher than that for TMS derivatives (Figure 1). Moreover, TMS derivatives degraded by 23.1% and 46.5% at 24 and 96 h post-reaction at room temperature, respectively, which was in contrast to the minimal degradation of TBDMS derivatives (Figure 1). This was consistent with the stability of silanides. More specifically, the steric hindrance effect of the TBDMS group can decrease the hydrolytic sensitivity and enhance the hydrophobicity and thermodynamic stability of derivatives [29,30]. Moreover, *t*-BDMCS resulted in an impurity peak near SA according to the chromatograms for MTBSTFA (Figure S1A,B). This finding was in line with the outcomes of previous research [31]. Therefore, MTBSTFA (97%) was selected. The mass spectrometry data of the SA, JA, and IAA derivatives are shown in Figure S2. However, some phytohormones, such as *trans*-zeatin riboside, gibberellic acid A3, and 24-epibrassinolide, are reportedly not suitable for an analysis involving MTBSTFA [32].

**Figure 2:**
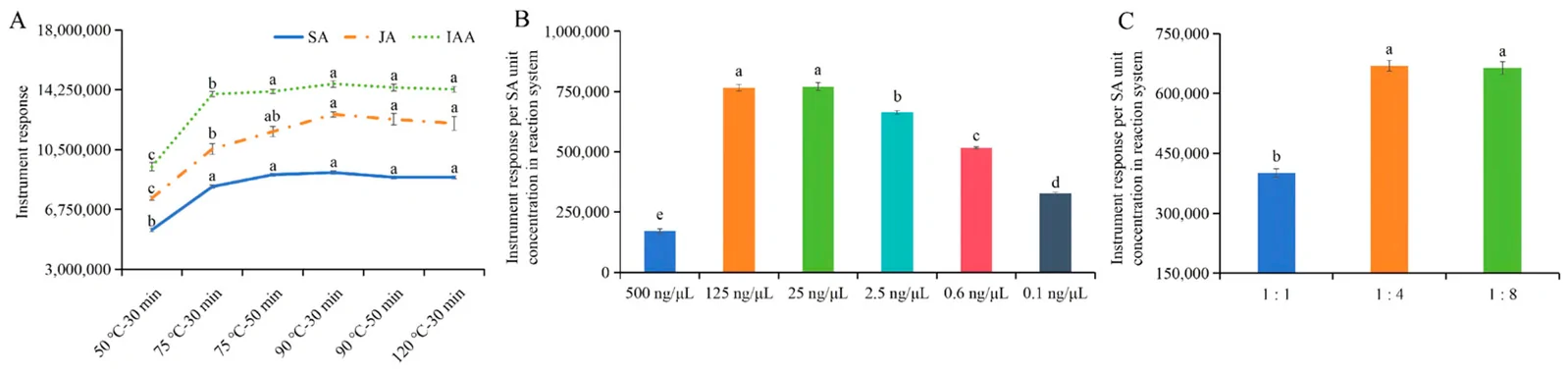
Effects of reaction conditions on the derivatization efficiency of MTBSTFA. (**A**) Reaction between a mixture comprising SA, JA, and IAA (8 ng µL^−1^ SA and 8 ng mL^−1^ JA and IAA; 50 µL) and 50 µL MTBSTFA at different incubation temperatures and times. (**B**) SA solutions with different concentrations were included in a reaction system (90 °C for 30 min) with an MTBSTFA-to-sample volume ratio of 1:8. (**C**) Different MTBSTFA-to-sample volume ratios were tested using 2.5 ng µL^−1^ SA solution at 90 °C for 30 min. Different letters indicate significant differences among treatments (Tukey’s test, *p* < 0.05). Data are presented as the mean ± standard error (*n* = 4).

Reaction conditions for MTBSTFA were optimized. Increasing the temperature can enhance derivatization efficiency by increasing the solubility of derivatization reagents and reactants [33]. The derivatization efficiency of three phytohormones increased significantly as the temperature increased up to 90 °C (Figure 2A). Notably, the derivatization efficiency was essentially unaffected by temperature increases from 90 to 120 °C or when the incubation time at 90 °C was extended to 50 min. Therefore, the optimized reaction conditions were 90 °C for 30 min. Reagent excess is important for silylation involving organic acids and α-keto acids, but derivatization efficiency decreases significantly if the MTBSTFA concentration is too high [34,35]. Similar results were obtained in the present study (Figure 2B). Moreover, when the SA solution concentration was 2.5 ng µL^−1^, which was close to the SA concentration in the tea leaf extract, the reaction efficiency was highest in the reaction system with an MTBSTFA-to-sample volume ratio of 1:4 (Figure 2C). Hence, to increase the reaction efficiency and conserve silylating agents, the optimal MTBSTFA-to-sample volume ratio was 1:4.

### 3.2. Optimization of Sample Purification

**Figure 3:**
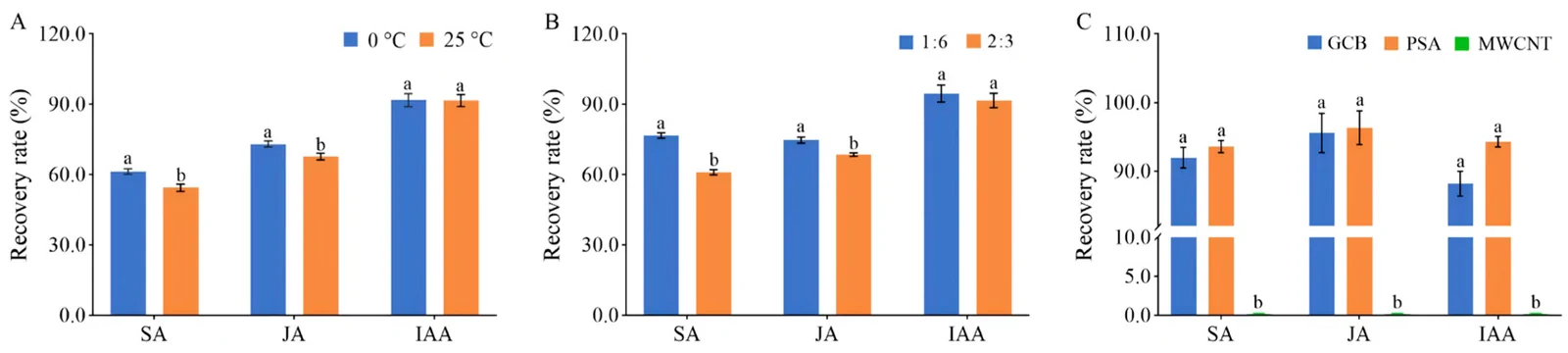
Effects of method parameters on the recoveries for MLLE and QuEChERS. (**A**) Different temperatures for MLLE (water-to-sample volume ratio of 2:3). (**B**) Different water-to-sample volume ratios for MLLE (0 °C). (**C**) Different adsorption materials for QuEChERS. A mixture comprising SA, JA, and IAA (8 ng µL^−1^ SA and 8 ng mL^−1^ JA and IAA) was used in three tests. Different letters indicate significant differences between or among treatments (*t*-test, *p* < 0.05 or Tukey’s test, *p* < 0.05). Data are presented as the mean ± standard error (*n* = 4).

MLLE and QuEChERS methods were used to purify the extract. Cooling in an ice bath and decreasing the water volume significantly enhanced the SA and JA recovery rates in MLLE (Figure 3A,B), likely because SA and JA are more soluble in water than IAA. For MLLE, the optimal condition was 100 µL water per 600 µL extract in an ice bath. The MLLE method was relatively simple because it could be performed in injection vials. Furthermore, it did not require substantial amounts of labor or reagents. In the QuEChERS step, three reverse-dispersive solid-phase adsorbents were compared. PSA was used to remove fatty acids, sugars, polar organic acids, lipids, and some pigments, whereas GCB was particularly effective for removing steroids and pigments. MWCNT effectively eliminated flavonoids, phenols, and organic acids because of their unique structure and relatively large surface area [36]. MWCNT strongly adsorbed three phytohormones (Figure 3C), in contrast to the extremely weak adsorption by PSA. Therefore, PSA was selected.

**Figure 4:**
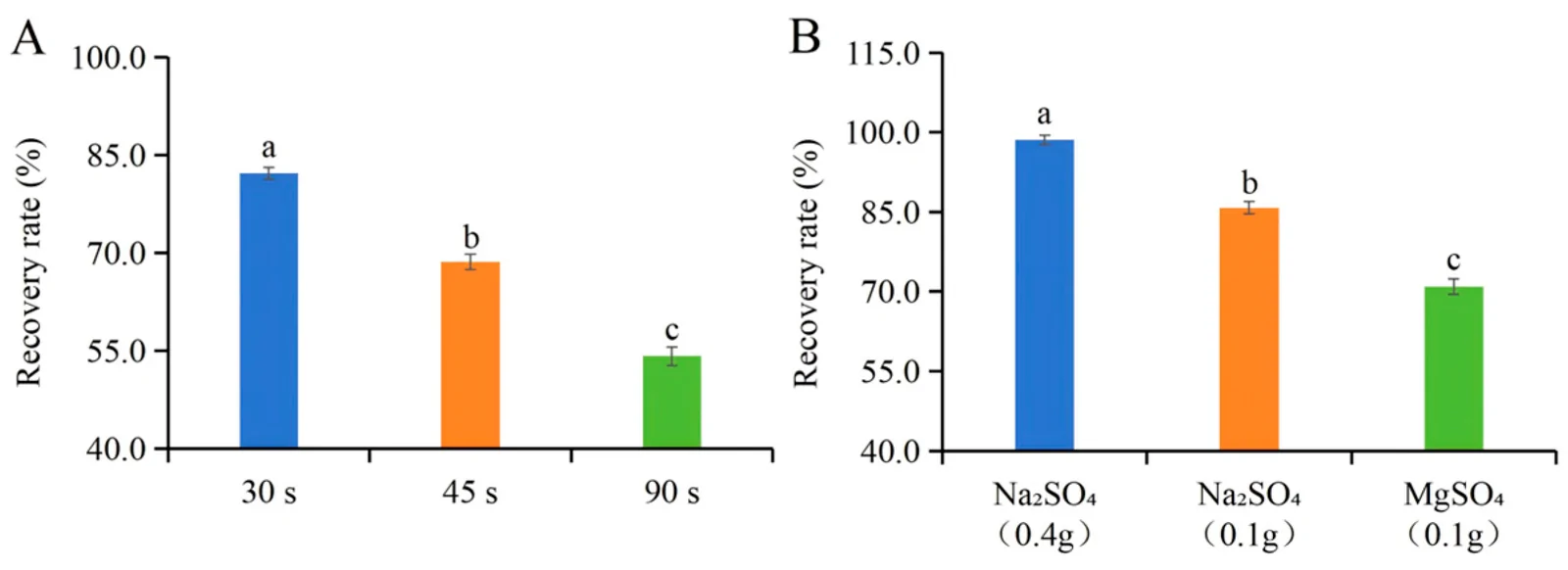
Dehydration effects of two methods. (**A**) Nitrogen blowing time (1.2 L min^−1^, 25 °C, and 200 µL sample). (**B**) Two dehydrating agents (400 µL sample). The test sample contained 8 ng µL^−1^ SA standard solution and 2% ultrapure water. Different letters indicate significant differences among treatments (Tukey’s test, *p* < 0.05). Data are presented as the mean ± standard error (*n* = 4).

Because silylating agents are sensitive to water, samples must be completely dry [32]. Usually, a nitrogen-blowing step is included to dry samples, but this leads to analyte loss (Figure 4A). In addition, precisely controlling the degree of nitrogen blowing was difficult. However, anhydrous sodium sulfate and anhydrous magnesium sulfate effectively removed moisture (Figure 4B), with anhydrous sodium sulfate eliminating moisture significantly better than anhydrous magnesium sulfate. Moreover, the recovery rate of 0.4 g anhydrous sodium sulfate was 98.6 ± 1.3% (Figure 4B). Because water is slightly soluble in ethyl acetate, 0.4 g anhydrous sodium sulfate was sufficient to remove water from 400 µL extracts. This dehydration step was combined with the QuEChERS method to simplify the purification procedure.

### 3.3. Sample Pre-Treatment for Metabolism Quenching

**Figure 5:**
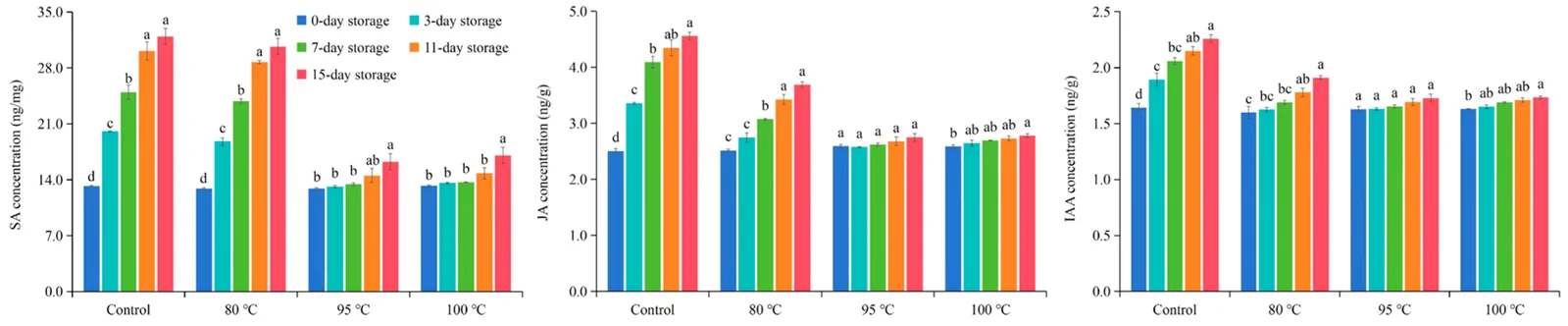
Effects of metabolism quenching on JA, SA, and IAA contents during storage at −80 °C. Freshly collected samples were immediately placed in hot water (80 °C for 10 min, 95 °C for 10 min, or 100 °C for 15 min). Samples that did not undergo the metabolism-quenching procedure were used as the control. Different letters indicate significant differences among treatments (Tukey’s test, *p* < 0.05). Data are presented as the mean ± standard error (*n* = 4).

**Figure 6:**
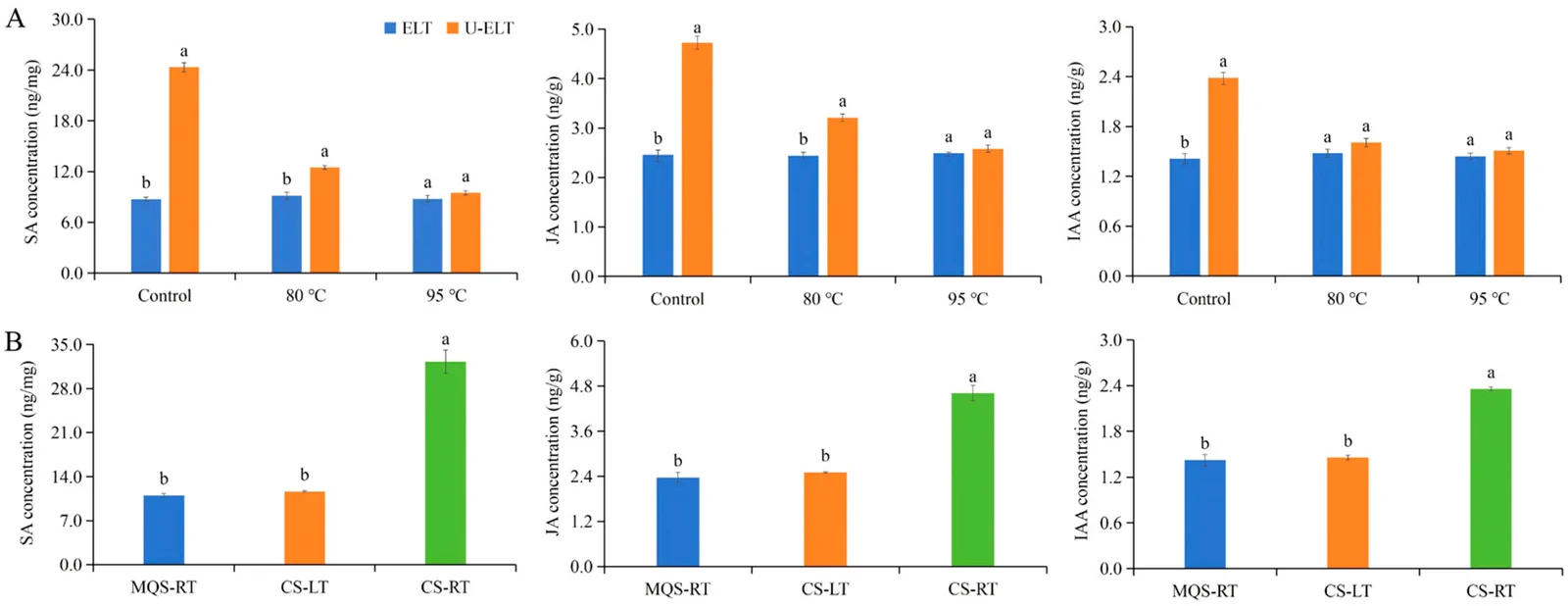
Effects of metabolism quenching on JA, SA, and IAA contents during the extraction. Freshly collected samples were immediately placed in hot water (80 °C for 10 min or 95 °C for 10 min). Samples that did not undergo the metabolism-quenching procedure were used as the control. (**A**) Metabolism-quenched and control samples were extracted at a low temperature (0 °C; ELT) or were left at room temperature (25 °C) for 20 min before being homogenized (U-ELT). (**B**) Control samples (CS) were extracted at a low temperature (LT) or at room temperature (RT), whereas metabolism-quenched samples (95 °C for 10 min, MQS) were extracted at RT. Different letters indicate significant differences between or among treatments (*t*-test, *p* < 0.05 or Tukey’s test, *p* < 0.05). Data are presented as the mean ± standard error (*n* = 4).

The composition of tissue extracts reflects the state of the tissue during sampling. In the present study, JA, SA, and IAA contents increased significantly as the duration of the storage at −80 °C was extended (Figure 5). SA, JA, and IAA contents increased significantly by 1.9, 1.6, and 1.3 times after a 7-day storage at −80 °C. We hypothesize that these phytohormones may be biosynthesized or converted from their corresponding derivatives (e.g., via hydroxylation, glycosylation, or methoxylation) even at ultra-low temperatures. However, considering there are no known reports regarding this phenomenon, the universality of this phenomenon and the underlying mechanism should be investigated. Additionally, the contents of the three analyzed phytohormones increased significantly when samples were left at room temperature for 20 min before being homogenized (Figure 6A) and when the extraction was conducted at room temperature (Figure 6B).

During storage and extraction, enzyme activities must be inhibited to prevent changes in phytohormone levels [37]. Appropriate metabolism quenching may prevent such undesirable changes. For example, a modified Bieleski’s solvent is appropriate for simultaneously extracting multiple phytohormones because it inhibits the enzymatic degradation of phytohormones [38,39,40]. Similarly, plant tissues used to examine phosphate metabolism should not be stored under cold conditions without inactivating phosphatase using methanol at high temperatures [41]. To maintain phytohormone levels during storage and extraction, a simple metabolism-quenching method was established in this study. More specifically, immersing samples in hot water prevented the increase in phytohormone levels during storage and extraction. There were no significant changes in SA, JA, or IAA contents in metabolism-quenched samples (incubated at 95 °C for 10 min or at 100 °C for 15 min) after a 7-day storage at −80 °C (Figure 5). Moreover, SA, JA, and IAA levels in metabolism-quenched samples (incubated at 95 °C for 10 min or at 100 °C for 15 min) did not change significantly, even if the samples were left at room temperature for 20 min before being homogenized (Figure 6A) or the extraction was performed at room temperature (Figure 6B). Therefore, samples were immersed in hot water (95 °C) for 10 min. This metabolism-quenching method simplified the analysis by eliminating the need for a low-temperature extraction, while also ensuring phytohormone data were accurate. Nevertheless, in the metabolism-quenched samples, the concentrations of phytohormones, especially SA, exhibited a marginal increase as the storage duration was prolonged at −80 °C. One possible explanation is that these phytohormones may be generated through the spontaneous decomposition of specific synthetic intermediates or derivatives. For example, isochorismate-9-glutamate, a key SA biosynthetic intermediate in *Arabidopsis thaliana*, can spontaneously decay into SA [42].

### 3.4. Method Validation

The quantification limits for SA, JA, and IAA standards were 0.004 ng µL^−1^, 0.003 ng mL^−1^, and 0.003 ng mL^−1^, respectively (Table S1). By contrast, quantification limits for tea leaves were 0.020 ng mg^−1^, 0.015 ng g^−1^, and 0.015 ng g^−1^, respectively. A comparison with UHPLC-MS/MS data revealed a decrease in the sensitivity for SA (40 times), but increases in the sensitivity for JA and IAA (3 and 7 times, respectively) [43,44]. Relatively linear changes in the contents of the three phytohormones were detected over four orders of magnitude (*R*^2^ > 0.999), with the smallest amounts close to the quantification limits (Table S1). This method had a good specificity for analyzing the three phytohormones in tea plant leaves (Figure S1C–E). Recoveries at three spiked levels (1/3, 2, and 6 times the basal levels) ranged from 93.1% to 98.9% (Table 1). The intra-day and inter-day reproducibility of the recovery was 2.1–9.9% and 3.2–8.3%, respectively (Table 1). According to these findings, 10 mg tea plant leaves were sufficient for simultaneous analyses of the three selected phytohormones. Hence, this method can reliably and accurately quantify SA, JA, and IAA contents in tea plants. Furthermore, it may be useful for analyzing spatial variations in the contents of these three phytohormones in a tea leaf.

Although MTBSTFA-based derivatization of phytohormones has been reported previously [32], phytohormone contents vary widely among plant species. Tea is a typical high-SA plant, in which the contents of SA, JA, and IAA differ by orders of magnitude. The derivatization system therefore required specific optimization. Moreover, tea leaves are rich in endogenous compounds, which cause substantial matrix interference. The efficiency of interferent removal during sample preparation is thus decisive for the feasibility of the method. Accordingly, the derivatization system and clean-up procedure were optimized in this study, enabling accurate quantification of the three phytohormones in tea plants by conventional GC-MS.

### 3.5. Spatiotemporal Changes in JA, SA, and IAA Contents in Damaged Tea Plants

**Figure 7:**
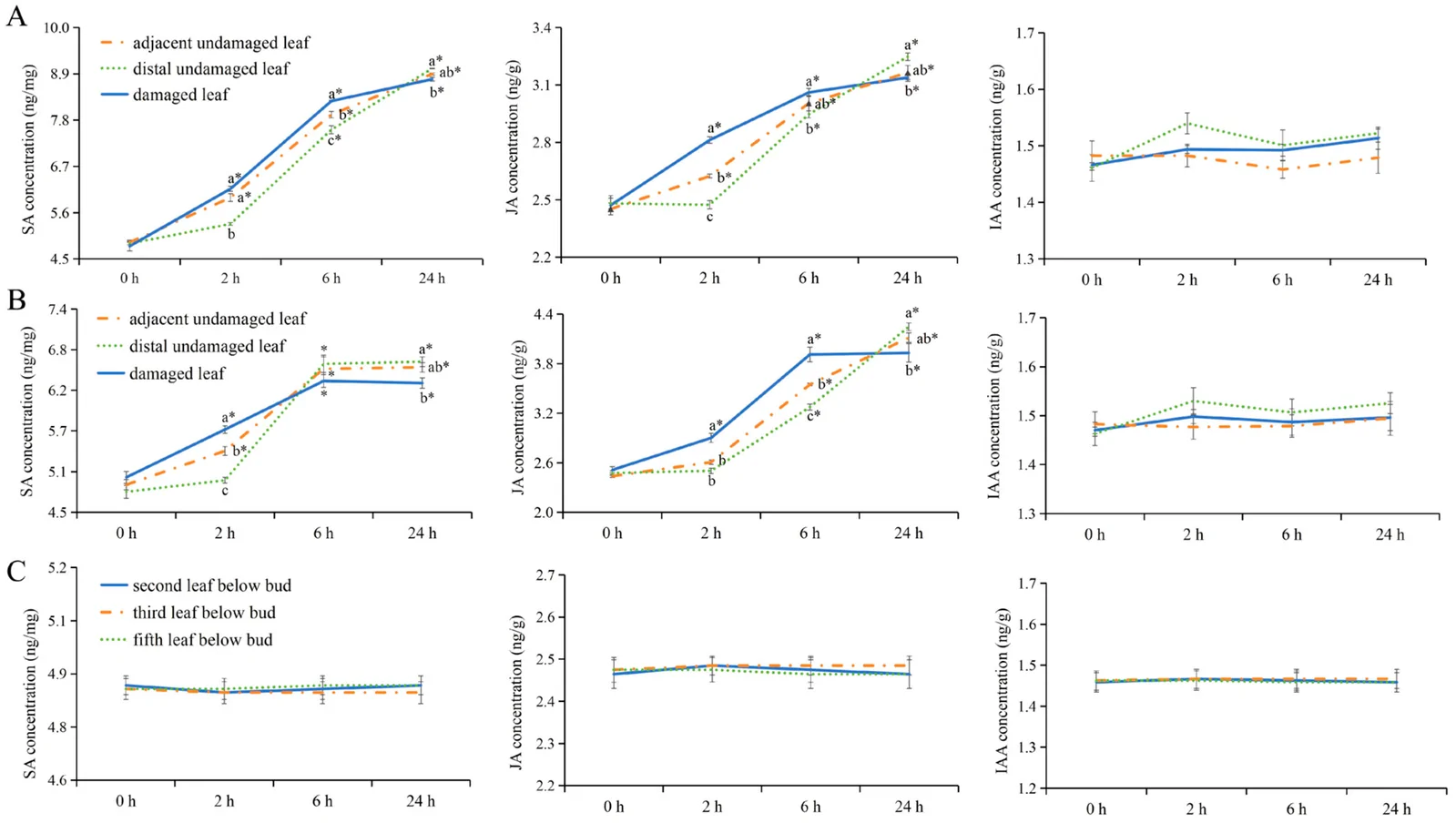
Local and systemic changes in JA, SA, and IAA contents in tea plants infested with leafhoppers (**A**) and geometrids (**B**). (**C**) Undamaged tea plants. Geometrid larvae or leafhopper nymphs were confined to the second leaf (damaged) below the bud. The first leaf (adjacent undamaged) and third leaf (distal undamaged) below the damaged leaf were used to evaluate the systemic response. Different letters indicate significant differences among damaged leaves, adjacent undamaged leaves, and distal undamaged leaves of damaged tea plants (Tukey’s test, *p* < 0.05). The asterisk indicates significant differences in leaves at the same position between damaged and undamaged tea plants (*t*-test, *p* < 0.05). Data are presented as the mean ± standard error (*n* = 4).

Using the method developed in this study, we determined the local and systemic changes in JA, SA, and IAA contents in tea plants infested with herbivorous insects. Leafhopper and geometrid infestations increased the contents of both SA and JA (Figure 7A,B). More specifically, in leaves damaged by geometrids, JA and SA contents increased 1.6 and 1.3 times, respectively, at 24 h post-infestation. Conversely, in the leafhopper treatment, JA and SA increased, respectively, 1.3 times and 1.8 times. Accordingly, the infestation by geometrids mainly induced JA accumulation, while the infestation by leafhoppers mainly induced SA accumulation. Whether these changes reflect actual activation of JA/SA signaling pathways requires further verification by measuring downstream signaling components. The observations in this study differed somewhat from those of an earlier study [26], in which leafhopper and geometrid infestations triggered an obvious increase in the JA content, but only a minor increase in the SA content. This inconsistency between studies may be related to the fact that detached tea shoots were used in the previous study [26]. Notably, the tea plant volatiles induced by the geometrids and leafhoppers are completely different [45]. The subtle differences in phytohormone content changes induced by geometrids and leafhoppers may not be sufficient to explain the substantial differences in the induced secondary metabolites. Moreover, compared with the volatiles induced by geometrids, the tea plant volatiles induced by leafhoppers were more similar to those induced by exogenous methyl jasmonate [45]. It is also possible that other phytohormones, or fine-tuning of JA and SA signaling, may contribute to the distinct metabolic responses. These possibilities require further investigation. For both geometrid and leafhopper infestations, SA and JA contents increased rapidly within the first 6 h. Next, the contents of these two phytohormones either increased slowly (leafhopper treatment) or remained almost unchanged (geometrid treatment) (Figure 7A,B). This difference may be associated with the diversity in feeding behaviors of geometrids and leafhoppers. In addition, the two pest infestations did not significantly affect the IAA content (Figure 7). This might be because the phytohormone contents were measured only within 24 h after infestation. The relatively short infestation duration may not be sufficient to trigger the balance between growth and defense in tea plants [46].

Rapid systemic changes in JA and SA contents were detected (Figure 7A,B). Specifically, JA and SA contents in the undamaged leaves adjacent to damaged leaves were generally significantly higher than the corresponding contents in the control plants at 2 h post-infestation. One possible explanation for this rapid systemic response is the involvement of induced volatiles, especially (Z)-3-hexenol, which is released immediately after leaf damage [45]. Earlier research indicated that this compound is an airborne signal that promotes the defense response of undamaged leaves on a damaged tea plant or an adjacent tea plant [47,48,49]. An airborne signal should be transmitted much faster than chemical signals in the xylem. JA and SA contents increased relatively slowly in leaves distal to damaged leaves in the first 2 h of the geometrid or leafhopper infestation. Moreover, systemic changes in JA and SA contents were generally significantly weaker than the corresponding local changes at 6 h post-infestation. However, at 24 h post-infestation, the systemic changes in JA and SA contents were significantly greater than the corresponding local changes (Figure 7A,B). These observations suggest that tea plants may coordinate local and systemic JA/SA responses to cope with herbivore infestation, although the underlying mechanism remains to be elucidated.

## 4. Conclusions

In this study, the GC-MS system was used to develop a highly sensitive and reliable method for analyzing JA, SA, and IAA in tea plants. A simple purification and a reliable silylation-based derivatization were completed before the GC-MS analysis. The sensitivity was similar to or exceeded that obtained by UHPLC-MS/MS. 10 mg tea leaves were sufficient for simultaneous analyses of the three phytohormones. This developed method should be a useful supplement to UHPLC-MS/MS methods. In particular, the SA, JA, and IAA contents in tea leaves increased significantly during storage at −80 °C and during extraction at room temperature. Therefore, a simple metabolism-quenching method was developed, which was also applicable to UHPLC-MS/MS analysis, to improve the accuracy and simplify the extraction. Finally, the developed method was used to determine the spatiotemporal changes in JA, SA, and IAA in tea plants damaged by two important pests with different feeding behaviors. Geometrid infestation induced a greater increase in JA than in SA, whereas leafhopper infestation induced a greater increase in SA than in JA. The systemic responses of JA and SA were rapid and similar to the local response. These results showed the proposed method was sensitive and reliable, and should enhance understanding of the phytohormone responses of tea plants to pest insects.

## Figures and Tables

**Table 1:** Recoveries and relative standard deviations (RSD) of JA, SA, and IAA from tea leaf samples.

Spiked Level	Compound	Recovery (%, *n* = 7)	Intraday RSD (%, *n* = 7)	Interday RSD (%, *n* = 7)
1/3 times basal levels	JA	97.7	5.7	5.2
	SA	96.7	6.3	6.9
	IAA	96.2	9.9	8.3
2 times basal levels	JA	93.1	4.0	4.0
	SA	97.1	5.2	4.0
	IAA	98.9	2.1	3.2
6 times basal levels	JA	95.9	4.4	4.8
	SA	97.5	3.2	4.9
	IAA	95.5	3.7	5.6

SA, JA, and IAA contents in the non-spiked control were 8.35 ± 0.09 ng mg^−1^, 2.54 ± 0.04 ng g^−1^, and 1.48 ± 0.02 ng g^−1^, respectively.

## Data Availability

All data supporting the findings of this study are available within the article and its Supplementary Materials. Further inquiries should be addressed to the corresponding author.

## Supplementary Materials

The following supporting information can be downloaded at: https://www.mdpi.com/article/10.3390/biology15201793/s1, Table S1: Linearity and sensitivity data for JA, SA, and IAA standards; Figure S1: Typical chromatograms for JA, SA, and IAA analyzed by GC-MS after a reaction with MTBSTFA; Figure S2: Mass spectra of SA, JA, and IAA analyzed by GC-MS after a reaction with MTBSTFA.
