# Supplementary material for: Analysis of Spatiotemporal Changes in Jasmonic Acid, Salicylic Acid, and Indole-3-Acetic Acid in Tea Plants Based on Silylation and Gas Chromatography-Mass Spectrometry
Source: Biology (Basel). 2026 Oct 9;15(20):1793. doi: 10.3390/biology15201793 (PMC13701311; doi:10.3390/biology15201793)
Supplement: Supplementary file 1 [file biology-15-01793-s001.zip › biology-4595849-supplementary.pdf]

Supplementary Materials

Table S1 Linearity and sensitivity data for JA, SA, and IAA standards

Compound	Ion (m/z)	Linearity rang	R^2 (n=3)	LOD (n=7)	LOQ (n=7)
SA	73, 309*	0.005-12.8 ng μL^{-1}	>0.999	0.001 ng μL^{-1}	0.004 ng μL^{-1}
JA	73, 265*	0.004-12.8 ng mL^{-1}	>0.999	0.001 ng mL^{-1}	0.003 ng mL^{-1}
IAA	75, 130, 232*	0.004-12.8 ng mL^{-1}	>0.999	0.001 ng mL^{-1}	0.003 ng mL^{-1}
IS	88, 101*	/	/	/	/

IS refers to the internal standard (ethyl decanoate). R^2 represents the correlation coefficient of calibration curves. LOD and LOQ represent detection and quantification limits, respectively, and are based on a signal 3 and 10 times higher than the standard deviation of the baseline noise. * indicates the quantitation ion, whereas / indicates a lack of information.

Figure S1

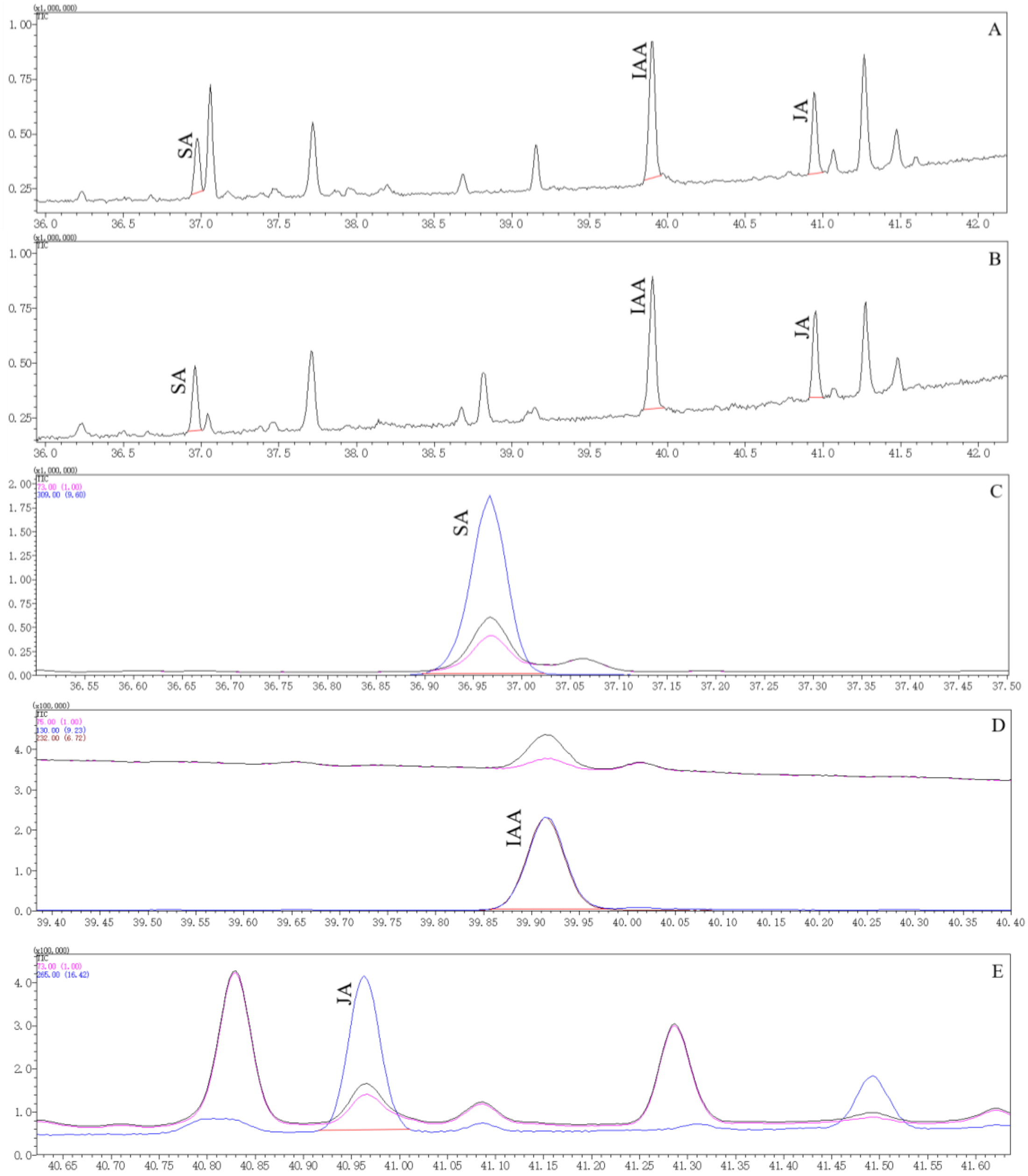

Figure S1 Typical chromatograms for JA, SA, and IAA analyzed by GC/MS after a reaction with MTBSTFA. A, Reaction between a mixture comprising SA, JA, and IAA (1 ng/µL SA and 1 ng/mL JA and IAA) and MTBSTFA with 1% *t*-BDMCS in the Scan mode. B, Reaction between the SA, JA, and IAA mixture and MTBSTFA in the Scan mode. Analysis of SA (C), IAA (D), and JA (E) contents in tea leaf samples using the optimized method and the selective ion monitoring mode.

Figure S2

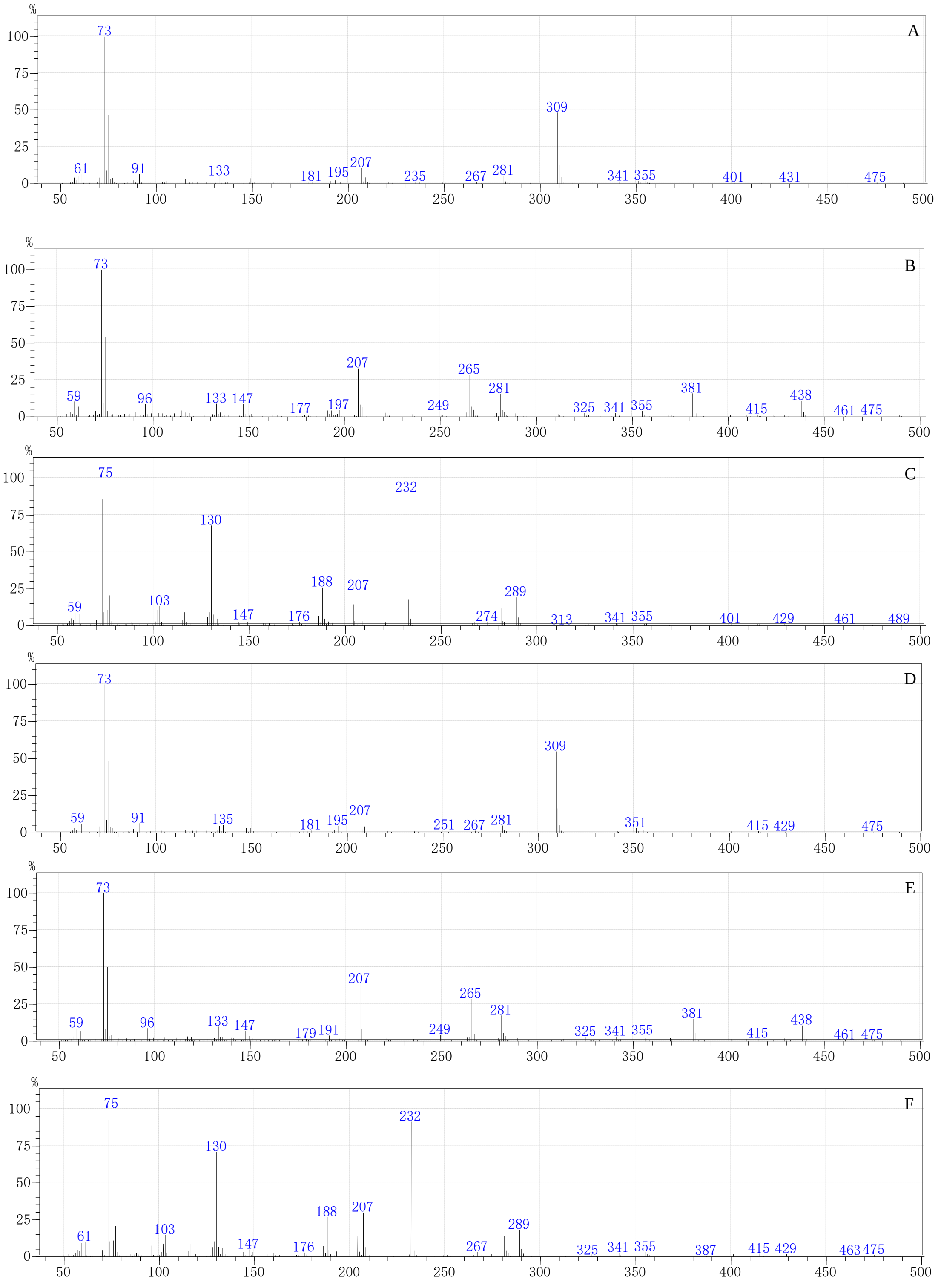

Figure S2 Mass spectra of SA, JA, and IAA analyzed by GC/MS after a reaction with MTBSTFA. A, SA standard; B, JA standard; C, IAA standard; D, SA in tea leaves; E, JA in tea leaves; F, IAA in tea leaves.
